# In vitro activity of *Houttuynia cordata* against bacteria isolated from diabetic foot

**DOI:** 10.1186/s41065-025-00505-5

**Published:** 2025-07-31

**Authors:** Chong Geng, Liuxuan An, Lin Niu, Xiaona Cui, Shulan Zhang, Xueqin Yuan, Qian Ma

**Affiliations:** Clinical Laboratory, Shijiazhuang Traditional Chinese Medicine Hospital, No.138, Jianhua Avenue, Chang’an District, Hebei, 050000 China

**Keywords:** Resistance to drugs, *Houttuynia cordata*, Infection, *Staphylococcus aureus*, *Pseudomonas aeruginosa*

## Abstract

**Background:**

Misuse of antibiotics makes it very easy for bacteria to become resistant to drugs. *Houttuynia cordata* (HC) has antibacterial, antiviral, analgesic, antioxidant, diuretic, hypoglycemic, and immune-enhancing properties.

**Aim:**

To study the inhibitory effect of HC on *Staphylococcus aureus* and *Pseudomonas aeruginosa* isolated from tissue or pus specimens from patients with diabetic foot ulcers.

**Materials and methods:**

Seventy-two patients with DFU were randomly divided into three groups and given treatment with methicillin (Met), meropenem (Mer), and HC, respectively. Analysis and identification of clinical isolates of *Staphylococcus aureus* and *Pseudomonas aeruginosa* using the Orbitrap Exploris™ 480. The drug susceptibility of four isolates to *HC* was studied by bacteriostatic test. The MIC and MBC of *Houttuynia cordata* against four isolates were determined using the broth microdilution method. The growth and time-kill curves of the bacteria were studied by bacterial inhibition experiments.The effect of *Houttuynia cordata* on the viability of H6C7 cells was examined by MTT assay.

**Results:**

HC treatment improved clinical parameters of DFUs patients. The inhibition zone of S08 (MSSA) was 15.45 ± 0.12 mm, which was highly sensitive to Houttuynia cordata. The inhibition zone sizes of R11 (MRSA), P10 (CRPA) and D22 (MDRPA) were 12.64 ± 0.09 mm,13.42 ± 0.11 mm and 11.23 ± 0.08 mm, respectively. All of them were moderately sensitive to Houttuynia cordata. The MIC of S08,R11,P10 and D22 were 31.25,62.5,62.5 and 125 µg/mL, respectively. The MBCS of S08,R11,P10 and D22 were 500,1000,1000 and 1000 µg/mLrespectively.Bacterial growth curves and time-kill curves demonstrated that *Houttuynia cordata* significantly inhibited the growth of both bacteria. Cytotoxicity assay showed that *Houttuynia cordata* effectively inhibited bacteria without cytotoxicity to eukaryotic cells.

**Conclusion:**

*Houttuynia cordata* exhibits promising antibacterial activity against both Staphylococcus aureus and Pseudomonas aeruginosa, including drug-resistant strains, without cytotoxic effects on eukaryotic cells. These findings support its potential as an alternative therapeutic agent for DFU-associated infections.

## Introduction

Diabetes Mellitus (DM) is one of the more common chronic metabolic diseases in clinical practice [1, 2]. The incidence of DM has increased in recent years [3, 4]. Diabetic foot ulcer (DFU) is one of the most serious complications of diabetes mellitus, and according to clinical statistics, about 30% of diabetic patients are at risk of contracting DFU [5, 6]. DFU are foot infections, ulcers, and deep tissue destruction due to abnormalities of the nerves and vascular lesions in the distal lower limbs of the patient’s feet [7, 8]. When an ulcer occurs various microorganisms take the opportunity to proliferate on the traumatized surface [9]. The proliferation of these bacteria can cause extensive tissue damage, inducing a series of inflammatory responses [10]. Even more serious is the fact that this secondary infection caused by the bacteria may put DFU patients at risk of cut-off or even death [11, 12]. Currently, microbiological testing is the mainstay of treatment for DFU infections in the clinical setting [13]. Many types of microorganism cause infections in patients with DFU, and the most commonly treated microorganisms are *Staphylococcus aureus* and *Pseudomonas aeruginosa* [14, 15]. The proliferation of these two bacteria may cause itching of the skin, redness and swelling of the foot skin, increased temperature of the foot skin, and other symptoms. In severe cases, there may also be symptoms such as acral ulcer and necrosis [16]. While modern research has yielded antibiotics against *Staphylococcus aureus* and *Pseudomonas aeruginosa*. There is insufficient evidence to support the idea that early use of broad-spectrum antibiotics promotes the healing of diabetic foot ulcers [17]. In addition, studies have shown that long-term application of antibiotics can lead to the development of resistance in the flora, making later treatment immeasurably more difficult [18]. Therefore, there is an urgent need to develop safe and effective new drugs to overcome this fight against bacterial infections.

*Houttuynia cordata* (*HC*), also known as Houttuynia cordata Thunb, is a dicotyledonous plant [19, 20]. *HC* is a relatively common plant in China, which not only has various medicinal components such as alkaloids, polysaccharides, brassicas, organic acids, and volatile oils, but also various nutrients [21, 22]. Some studies have shown that *HC* has antibacterial, antiviral, analgesic, antioxidant, diuretic, hypoglycemic, and immune-enhancing properties [19, 23]. In addition, *HC* has been clinically reported for the treatment of pulmonary infections [24], but there are no reports related to its use for diabetic foot ulcer infections. Therefore, the study of the bacteriostatic effect of *HC* can provide a scientific basis for the treatment of clinical diabetic foot ulcers and is of great significance for the development of bacteriostatic products of *HC*.

This study aimed to investigate the inhibitory effect of *HC* on four strains of bacteria isolated from tissue or pus specimens from patients with diabetic foot ulcers. We determined the MIC and MBC of *HC* against four isolates using broth microdilution method. The bacteriostatic effect of *HC* was demonstrated by the study of bacterial growth curves and time-kill curves. The cytotoxicity of *HC* on eukaryotic cells was demonstrated by studies of H6C7 cell activity.

## Material and method

### Clinical cases

A total of 84 DFUs patients were recruited in this study between May 2024 and October 2024, and a total of 72 patients met the inclusion and exclusion criteria. They were then randomly assigned to receive methicillin (Met, *n* = 24), meropenem (Mer, *n* = 24) or *Houttuynia cordata* extract (*HC*, *n* = 24). Inclusion criteria: [1] Patients aged between 30 and 70 years old [2]. The patient had a definite history of diabetes mellitus with long-term poor blood glucose control [3]. The patient’s fasting blood glucose was ≥ 7.0 mmol/L and 2-hour postprandial blood glucose was ≥ 11.1 mmol/L [4]. The patient had an elevated white blood cell count and C-reactive protein [5]. The patient has a Wagner classification of 1–2. Exclusion Criteria: [1] Failure to obtain informed consent from patients and their families [2]. Poor patient adherence to treatment [3]. Patients with other malignant diseases [4]. Patients with non-diabetic lower extremity ulcers (e.g., traumatic, varicose vein ulcers). This clinical trial was conducted following approval by the Institutional Ethics Committee of Shijiazhuang Traditional Chinese Medicine Hospital (Approval No. 20250714056). Written informed consent was obtained from all participants prior to enrollment. The approval date is July 14, 2025.

### Preparation of *HC* extract

*Houttuynia cordata* (HC) tablets (50 g), obtained from a certified herbal pharmacy, were soaked in 500 mL of distilled water for 2 h at room temperature. The mixture was then boiled at 100 °C for 30 min. The residue was collected, soaked again using the same procedure, and both extracts were combined. The solution was filtered through a 0.45 μm membrane filter, centrifuged at 5,000 rpm for 10 min, and concentrated to 50 mL using a rotary evaporator (RE-2000 A, China). The resulting extract was adjusted to a final concentration of 1 mg/mL. The solution was sterilized by autoclaving at 121 °C for 15 min, then diluted to various working concentrations using a 2-fold serial dilution method. The extraction protocol was adapted from [25]. This extraction protocol was modified by us because we studied the effects of *Houttuynia cordata* extract at high concentrations on diabetic foot. Therefore, we increased the dosage of HC. Such that we obtained the concentration of HC extract in the order 500 µg /mL, 250 µg /mL 125 µg /mL, 62.5 µg /mL, 31.2 µg /mL, 15.6 µg /mL, 7.8 µg /mL, 3.9 µg /mL, 1.95 µg/mL.

### Preparation of drug sensitive tablets

Qualitative filter paper was made into 6 mm round small paper pieces. Subsequently, 30 of these paper discs were placed into a triangular flask. Following autoclaving for 15 to 20 min, the samples were dried and stored for further use. Thereafter, 0.25 mL of HC extract was added to the triangular flask containing the previously prepared small round filter papers. The small round filter papers were allowed to fully absorb the extract and were then dried for subsequent experimentation.

### Treatment of patients with DFUs

Patients with diabetic foot ulcers (DFUs) were randomly assigned into Met (*n* = 24) group, Mer (*n* = 24) group and (HC, *n* = 24) group to receive different treatments. The Met group received oral methicillin 0.5 g three times daily, while the Mer group received intravenous meropenem 0.5 g once daily. Treatment lasted for 14 days. Throughout the treatment period, patients were routinely monitored for inflammatory parameters and microbiological assessments. Patient confidentiality was maintained in accordance with ethical guidelines.

### Detection of TNF-α and IL-6 by ELISA

120 µl antibodies for TNF-α (ab285312) and IL-6 (ab233706) were added to 96-well plates. The cells were incubated at 37 °C for 2 h. Then, a 3% solution of BSA was added and incubated for 1 h at 37 °C to block non-specific binding sites. Next, serum from the three groups of patients was added to each well. Hrp-labeled streptavidin (diluted at a ratio of 1:2000) was added to the samples, followed by incubation at 37 °C in the dark for 30 min. Finally, 100 µL of TMB substrate was added to each well, followed by incubation in the dark for 10 to 15 min to allow color development. The absorbance values were subsequently measured at a wavelength of 450 nm using Varioskan LUX multimode microplate reader (ThermoFisher, USA).

### Bacterial isolation and antimicrobial testing

Bacterial isolation and identification 50 strains of *Staphylococcus aureus* and 50 strains of *Pseudomonas aeruginosa* were isolated from tissue or pus specimens retained after local debridement of patients with diabetic foot ulcers in our vascular unit. After identification by a mass spectrometer Orbitrap Exploris™ 480 (Thermo Fisher), we selected four isolates each of methicillin-sensitive *Staphylococcus aureus* (MSSA), methicillin-resistant *Staphylococcus aureus* (MRSA), carbapenem-resistant *Pseudomonas aeruginosa* (CRPA), and multidrug-resistant *Pseudomonas aeruginosa* (MDRPA).

### Preparation of bacterial inoculum

The experimental strains were passaged 2–3 times on plates containing basal medium, and then colonies were inoculated into Mueller-Hinton broth (MH broth) medium (Thermo Scientific™) by using an inoculating loop. The strains were incubated in a constant temperature incubator at 37℃ for overnight culture. The cell concentration was corrected with saline to 0.5 McFarland turbidity units. After dilution according to 1:100, the bacteria were inoculated within 15 min to obtain a suspension of 1.5 × 10^6^ CFU/mL.

Antibacterial assay (Disk diffusion method)The 15 mL of *HC* extract was placed in a clean sterile test tube. The 15 sensitized tablets were immersed in a test tube for 24 h. The tablets were dried under sterile conditions. 40 µL of bacterial suspension were prepared at a concentration of 1.6 × 10^6^ CFU/mL. The samples were spread evenly on agar plates. Then, the sensitized tablets were applied to the corresponding plates. The above plates were incubated at 37 °C for 24 h and the diameter of the circle of inhibition was measured.

### Minimum inhibitory concentration (MIC) assay

The *HC* extracts were double diluted. The diluted *HC* extracts were added to 96-well plates respectively. The drug concentration in each well was 1000 µg/mL, 500 µg /mL, 250 µg /mL 125 µg/mL, 62.5 µg/mL, 31.2 µg/mL, 15.6 µg/mL, 7.8 µg/mL, 3.9 µg /mL, 1.95 µg/mL respectively. The 11th well without *HC* was used as a growth control. 100 µL of bacterial suspension was added to each well. The 96-well plate was then incubated in a constant temperature incubator at 37 °C for 24 h. The minimum drug concentration without turbidity change was used as the MIC of *HC*.

### Minimum bactericidal concentration (MBC) assay

The 40 µL of liquid from the wells that did not undergo turbidity changes was inoculated in the MH solid. The plates were incubated at 37 °C for 24 h. The minimum drug concentration corresponding to a colony number less than 5 was used as the MBC for *HC*.

### After obtaining the MIC and MBC of the four bacteria, the growth curves, time kill kinetics assay and cytotoxicity of HC assay further demonstrated that HC had a better effect on relieving diabetic foot than antibiotics

#### Effects of *HC* on growth curves

To investigate the effects of antibiotics commonly used in clinical practice on two bacteria. The above configured MSSA (S08) and MRSA (R11) suspensions at a final concentration of 1.6 × 10^6^ CFU/mL were treated with 0 µg/ml and 50 µg/ml of methicillin (Met), respectively. Similarly, CRPA (P10) and MDRPA (D22) suspensions were treated with 0 µg /ml and 50 µg /ml of Meropenem (Mer), respectively. To investigate the effect of *HC* on two bacteria. Four isolates of MSSA (S08), MRSA (R11), CRPA (P10), and MDRPA (D22) were titrated serially at concentrations of 0,1.95,3.90, 7.80, 15.60, 31.20, 62.50, 125, 250, 500 and 1000 µg/ml of *HC*, respectively. Samples were taken at 1 mL every 4 h. Finally, the OD values were determined at 600 nm.

#### Time kill-kinetics assay

Suspensions of the above four isolates were prepared into 0.5 McCloud turbid units. Each isolate was then inoculated into an MH broth medium. The concentration of the final bacterial suspension was 1.6 × 10^6^ CFU/mL. Then, the highest dilution concentration was twice the MIC of *HC*. *HC* extracts of 0×MIC, 1/4×MIC, 1/2×MIC, 1×MIC, and 2×MIC were added sequentially to the test tubes of each isolate. The tubes were incubated at 37 °C for 24 h. Then we sampled every four hours. 100 µL of the bacterial suspension was spread onto MH agar plates. The MH agar plates were incubated at 37 °C for 24 h. Finally, the growth of colonies on each plate was detected by the CFU viable bacteria counting method.

#### Cytotoxicity of *HC* assay

Log-phase human normal pancreatic ductal epithelial cells (H6C7) cells were seeded at 1 × 10^4^ cells per well in 96-well plates.After the cells were spread over the bottom of the wells, H6C7 cells were treated with 15.6, 31.2, 62.5, 125, 250 µg/mL of *HC* for 24 h. Then 20 µL of 0.5% MTT solution was added to each well. The 96-well plate was continued to be incubated at 37 °C, 5% CO_2_ for 4 h. After terminating the incubation, 15 µL of DMSO was added per well, and then the crystals were fully dissolved by shaking at low speed for 10 min. Finally, the absorbance value of each well was determined at 490 nm.

### Statistical analysis

Data are presented as (Mean ± SD). Two-way analysis of variance (ANOVA) or one-way Analysis of Variance were performed. *p* < 0.05 was statistically significant.

## Results

### Changes in clinical parameters of DFUs patients after HC treatment

**Fig. 1:**
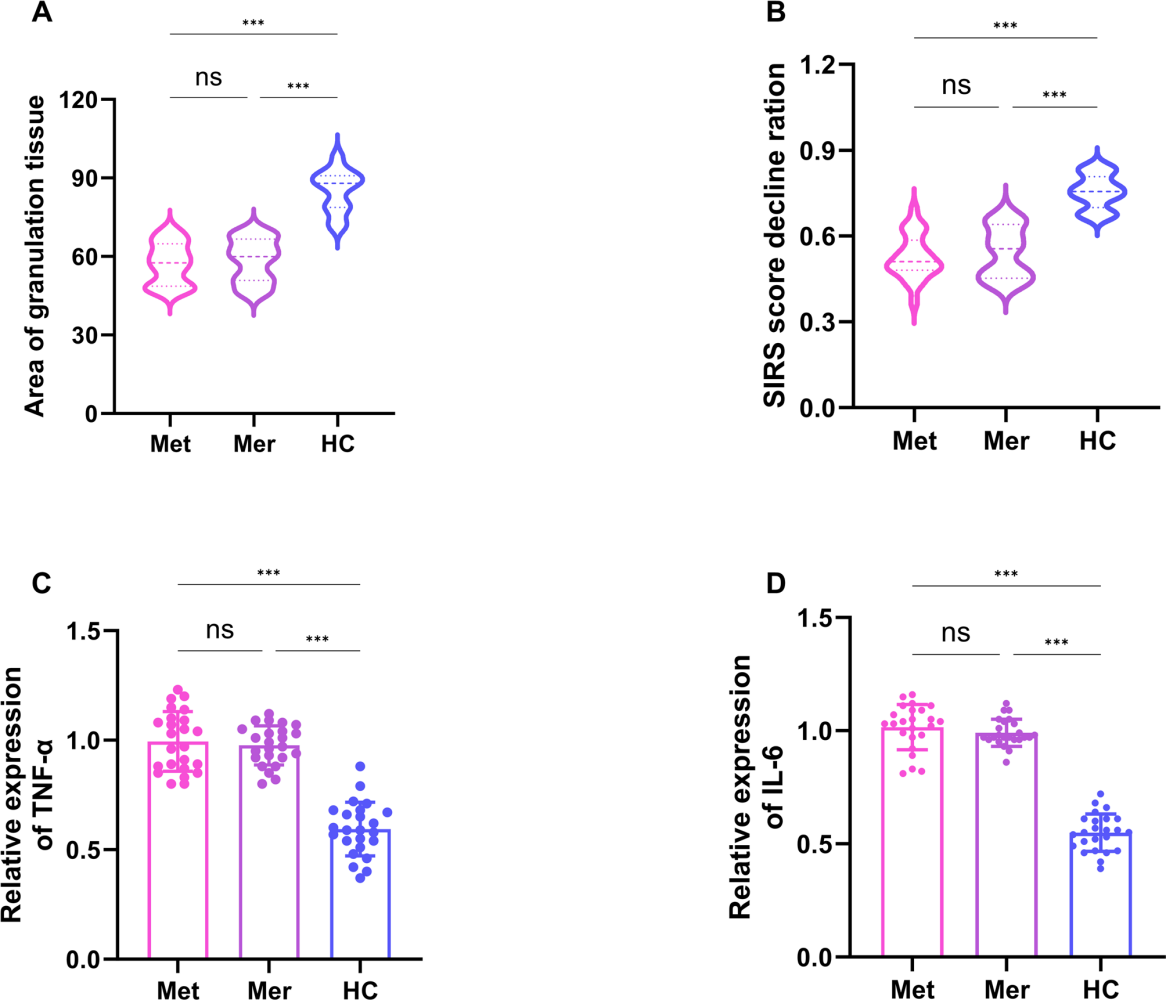
Changes in clinical parameters of DFUs patients after HC treatment. **(A)** After 14 days of treatment, the quantitative analysis of the granulation tissue (Met group *n* = 24, Mer group *n* = 24, HC group *n* = 24). **(B)** After 14 days of treatment, the quantitative analysis of the SIRS score decline ratio (Met group *n* = 24, Mer group *n* = 24, HC group *n* = 24). **(C-D)** After 14 days of treatment, ELISA was used to measure the serum levels of TNA-α and IL-6 (Met group *n* = 24, Mer group *n* = 24, HC group *n* = 24)

We compared and analyzed the differences of clinical information among the three groups. Table 1 shows that there were no statistically significant differences in Age (y), Body Mass Index (BMI) (kg/m2), Duration of ulcer (d), White Blood Cell (WBC) (×10^9^), C-Reactive Protein (CRP) (mg/L), Systemic Inflammatory Response Syndrome (SIRS) score, IL-6 (pg/ml), TNF-α (pg/ml), TG (mmol/L) and TC (mmol/L) of the three groups patients. Figure 1A illustrates that wound infection was quantified and measured over the course of 14 days of treatment. We found that the Area of granulation tissue was significantly higher in the HC group than in the Met and Mer groups(*p* < 0.001). Figure 1B illustrates that the Skin Infection Rating Scale score results showed that the SIRS score decline ration was higher in the HC group than in the Met and Mer groups (*p* < 0.001). In addition, Fig. 1C-D illustrates that the expression levels of TNF-α and IL-6 in the HC group were significantly lower than those in the Met and Mer groups after 14 days of treatment (*p* < 0.001). These resultsindicated that HC may effectively improve the infection of DFU patients.

**Table 1:** Baseline data of DFU patients

factors	Met (*n* = 24)	Mer (*n* = 24)	HH (*n* = 24)	*p*
Age (y)	51.43 ± 9.91	55.88 ± 7.58	54.95 ± 8.90	0.176
BMI (kg/m2)	21.80 ± 3.88	23.02 ± 4.44	23.03 ± 3.38	0.458
Duration of ulcer (d)	51.95 ± 10.08	54.66 ± 7.52	51.77 ± 11.60	0.528
WBC (×109)	15.29 ± 4.16	13.31 ± 1.83	15.33 ± 4.26	0.092
CRP (mg/L)	70.62 ± 1.72	68.92 ± 10.20	69.23 ± 12.39	0.787
SIRS score	7.06 ± 3.18	6.34 ± 2.49	8.07 ± 2.84	0.115
IL-6 (pg/ml)	84.52 ± 21.40	68.47 ± 22.99	73.61 ± 9.38	0.074
TNF-α (pg/ml)	77.39 ± 14.05	85.19 ± 18.60	75.38 ± 14.10	0.081
TG (mmol/L)	1.25 ± 0.33	1.01 ± 0.65	1.26 ± 0.55	0.194
TC (mmol/L)	2.77 ± 0.56	3.41 ± 0.85	2.66 ± 0.59	0.273

### Effect of *HC* on the circle of Inhibition

Table 2 shows that the diameter of the circle of inhibition of S08 (MSSA) was 15.45 ± 0.12 mm. Therefore, we determined that S08 (MSSA) was highly sensitive to *HC*. In addition, the diameter of the ring of inhibition for R11 (MRSA) was 12.64 ± 0.09 mm, the diameter of the ring of inhibition for P10 (CRPA) was 13.42 ± 0.11 mm and, the diameter of the ring of inhibition for D22 (MDRPA) was 11.23 ± 0.08 mm. Therefore, we judged the three isolates to be moderately sensitive to *HC*.

**Table 2:** Bacteriostatic test of *Houttuynia cordata*

serial number	strain type	inhibition circle diameter (mm)
S08	methicillin-sensitive S*taphylococcus aureus* (MSSA)	15.45 ± 0.12
R11	methicillin-resistant S*taphylococcus aureus* (MRSA)	12.64 ± 0.09
P10	carbapenem-resistant *Pseudomonas aeruginosa* (CRPA)	13.42 ± 0.11
D22	multidrug-resistant *Pseudomonas aeruginosa* (MDRPA)	11.23 ± 0.08

### MIC and MBC of *HC*

We evaluated the inhibitory activity of *HC* against four isolates and the results are shown in Table 3. Table 3 shows that the MIC for S08 (MSSA) was 31.25 µg /mL and its MBC was 500 µg /mL. The MIC for R11 (MRSA) was 62.5 µg /mL and its MBC was 1000 µg /mL. The MIC for P10 (CRPA) was 62.5 µg /mL and its MBC was 1000 µg /mL. The MIC for D22 (MDRPA) was 125 µg /mL and its MBC was 1000 µg /mL. In summary, we have come to the preliminary conclusion that *HC* can inhibit the growth of the above four isolates.

**Table 3:** Minimum inhibitory concentrations (MIC) and minimum bactericidal concentrations (MBC) of *Houttuynia cordata* against drug-resistant bacterial species

serial number	strain type	MIC (µg/mL)	MBC (µg/mL)
S08	methicillin-mensitive *Staphylococcus aureus* (MSSA)	31.25	500
R11	methicillin-resistant *Staphylococcus aureus* (MRSA)	62.5	1000
P10	carbapenem-resistant *Pseudomonas aeruginosa* (CRPA)	62.5	1000
D22	multi-drug-resistant *Pseudomonas aeruginosa* (MDRPA)	125	1000

### Growth curves demonstrate the inhibitory effect of *HC* on bacteria

**Fig. 2:**
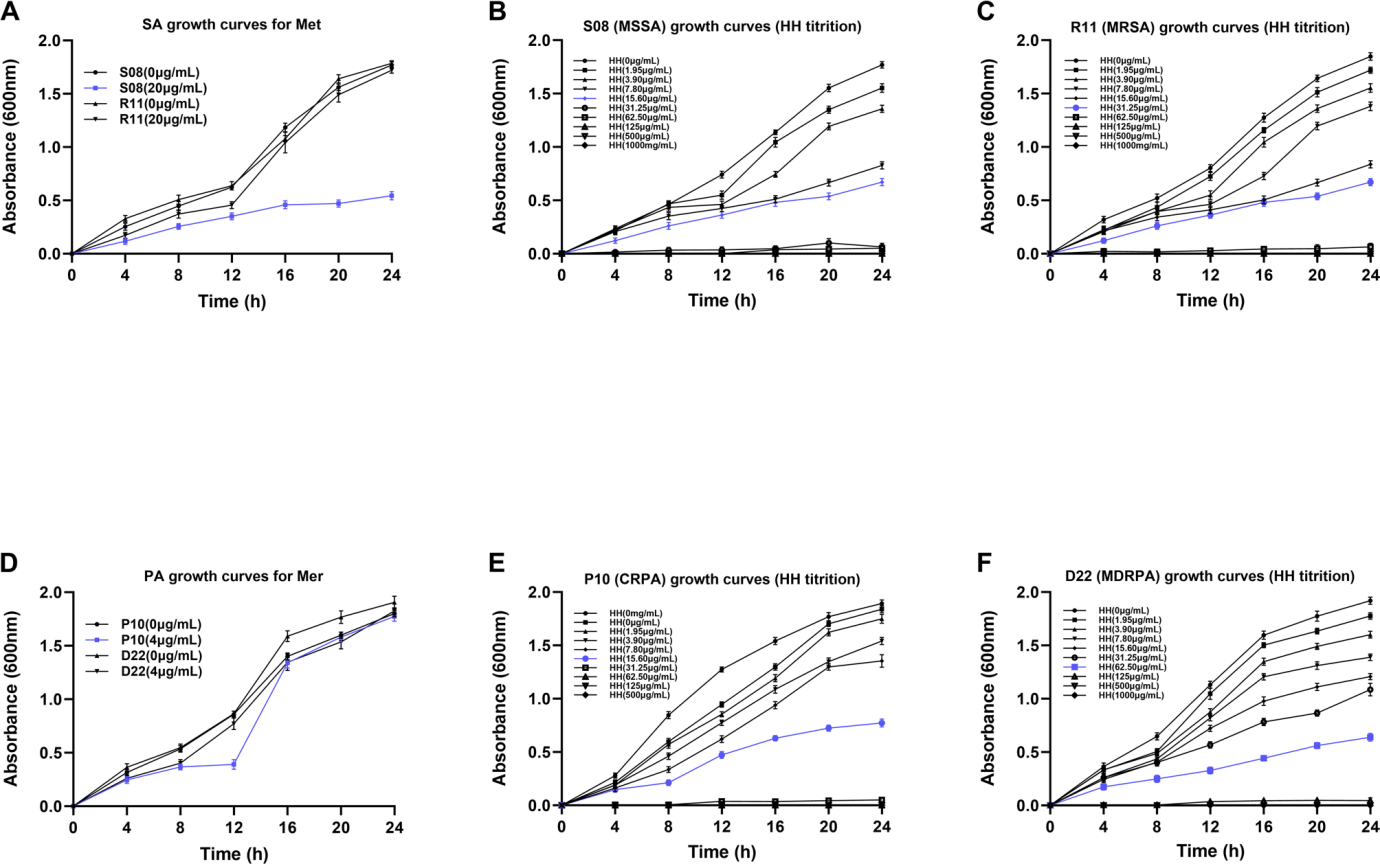
Effect of *Houttuynia cordata* on the circle of inhibition. **(A)** Effect of methicillin on the growth curves of S08 (MSSA) and R11 (MRSA). Effect of different concentrations of *Houttuynia cordata* extracts on the growth curves of S08 **(B)** and R11 **(C)**. **(D)** Effect of meropenem on the growth curves of P10 (CRPA)and D22 (MDRPA). **(E**,** F)** Effect of different concentrations of *Houttuynia cordata* extracts on the growth curves of P10 **(E)** and D22 **(F)**. The error bars are standard errors for three independent experiments

The Orbitrap Exploris™ 480 mass spectrometry analysis showed that S08 was a methicillin-sensitive *Staphylococcus aureus* (MSSA). However, R11 was a methicillin-resistant *Staphylococcus aureus* (MRSA). Therefore, we investigated the effect of methicillin on the growth curves of two strains, S08 and R11. Figure 2A illustrates that methicillin could inhibit the growth of S08 (MSSA) to some extent within 24 h. However, the inhibitory effect of methicillin on R11 (MRSA) was not satisfactory. Figure 2A illustrates that that the growth of R11 was significantly inhibited during the first 12 h of treatment with methicillin. However, after 12 h, R11 began to proliferate rapidly. Interestingly, titration experiments with *HC* showed that *HC* effectively inhibited the growth of S08 (MSSA) and R11 (MRSA). Figure 2B and C ilusstrates that different concentrations of *HC* inhibited both S08 (MSSA) and R11 (MRSA) to some extent, and the greater the concentration the stronger the inhibitory effect. Figure 2B illustrates that at a concentration of *HC* of 31.25 µg /mL, S08 (MSSA) showed almost no growth. Figure 2C illustrates that at a concentration of *HC* of 62.5 µg /mL, R11 (MRSA) showed almost no growth. Similarly, we studied the growth curves of two resistant strains of *Pseudomonas aeruginosa* using the above method. The Orbitrap Exploris™ 480 mass spectrometry analysis showed that P10 was Carbapenem-Resistant *Pseudomonas aeruginosa* (CRPA). However, D22 was a Multi-Drug-Resistant *Pseudomonas aeruginosa* (MDRPA). Therefore, we investigated the effect of meropenem on the growth curves of two strains, P10 and D22. Figure 2D illustrates that the growth of both P10 (CRPA) and D22 (MDRPA) was inhibited in the fore 8 h after meropenem treatment. However, from 12 h onwards, both P10 (CRPA) and D22 (MDRPA) began to proliferate rapidly. The growth at 24 h was almost completely unaffected by meropenem. Notably Fig. 2E and F illustrates that *HC* significantly inhibited the growth of P10 (CRPA) and D22 (MDRPA), and the inhibition was stronger at higher concentrations. In addition, Fig. 2E illustrates that almost no growth of P10(CRPA) when the concentration of *HC* was 62.5 µg /mL. Figure 2F illustrates that at 125 µg /mL, there was almost no growth of D22 (MDRPA). Therefore, we conclude that *HC* inhibits the growth of four strains S08 (MSSA), R11 (MRSA), P10 (CRPA), and D22 (MDRPA).

### Effect of *HC* on the time-kill curve

**Fig. 3:**
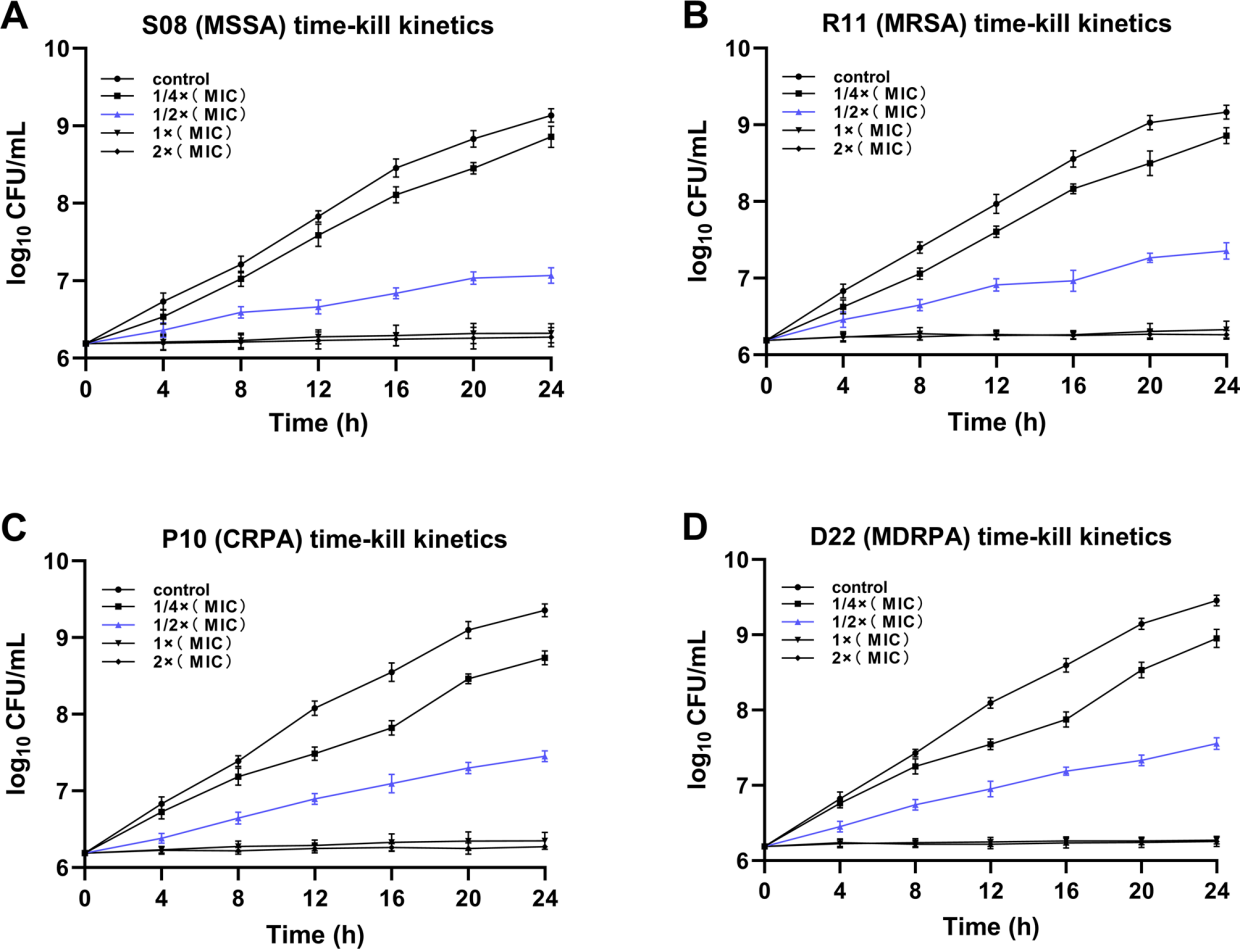
Effect of *Houttuynia cordata* on the time-kill curve. The time-kill curve for S08 (MSSA) **(A)** R11 (MRSA) **(B)**, P10 (CRPA) **(C)**, and R11 (MRSA) **(D)**. The error bars are standard errors for three independent experiments

To further demonstrate the inhibitory effect of *HC* on the above four isolates. We investigated their time-killing kinetics. Figure 3A illustrates thatdifferent concentrations of *HC* had different bacterial inhibition efficiencies, and the higher the concentration the higher the rate of bacterial inhibition. In addition, Fig. 3A illustrates that the concentration of *HC* at 1 × MIC (31.25 µg /mL) and above completely inhibited the growth of S08 (MSSA). Figure 3B illustrates that the concentration of *HC* at 1 × MIC (62. 5 µg /mL) and above completely inhibited the growth of R11 (MRSA). Similarly, for *Pseudomonas aeruginosa*, Fig. 2C illustrates that the same trend. The concentration of *HC* at 1 × MIC (62. 5 µg /mL) and above completely inhibited the growth of P10 (CRPA). Figure 3D illustrates that the concentration of *HC* at 1 × MIC (125 µg /mL) and above completely inhibited the growth of D22 (MDRPA). The results of the time-kill curves once again demonstrated that *HC* could effectively inhibit the growth of the four isolates.

### Cytotoxicity of *HC*

**Fig. 4:**
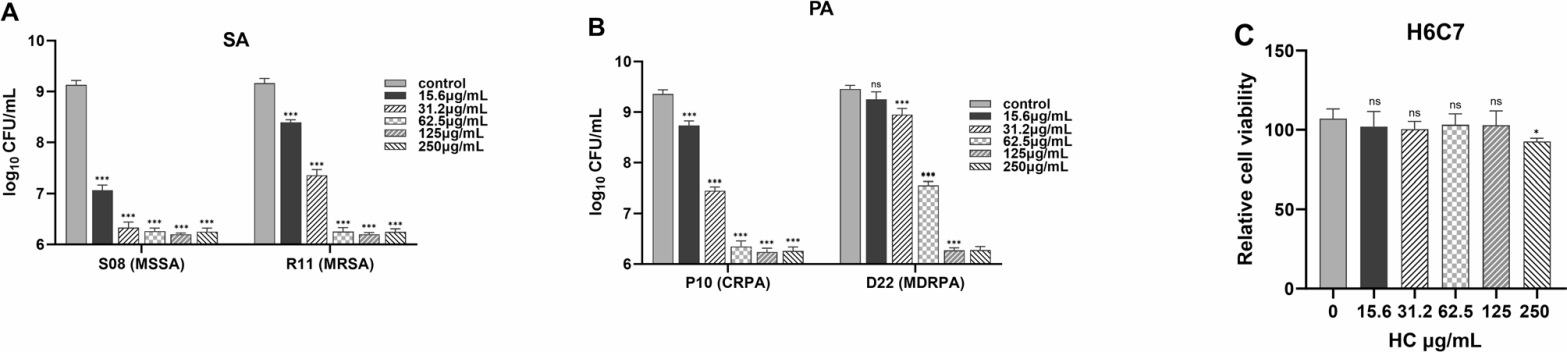
Cytotoxicity of *Houttuynia cordata*. **(A)** Growth of S08 (MSSA) and R11 (MRSA) detected by CFU viable bacteria counting method. **(B)** Growth of P10 (CRPA) and D22 (MDRPA) detected by CFU viable bacteria counting method. **(C)** Using the MTT assay to detect the effect of *HC* on the viability of H6C7 cells. *p < 0.05 vs. control. ***p < 0.001 vs. control

We examined the effect of *HC* on H6C7 cell viability using the MTT assay. Firstly, Fig. 4A illustrates that log_10_CFU/mL of S08 (MSSA) was significantly reduced when the concentration of *HC* was 31.25 µg /mL; while the log_10_CFU/mL of R11 (MRSA) was significantly reduced at 62.5 µg /mL(*p* < 0.001). Similarly, Fig. 4B illustrates that the log_10_CFU/mL of P108(CRPA) was significantly reduced when the concentration of *HC* was 62.5 µg /mL; while the log_10_CFU/mL of D22 (MDRPA) was significantly reduced at 125 µg/mL (*p* < 0.001). Excitingly, Fig. 4C illustrates that *HC* concentrations at 125 µg/mL and below did not affect the cellular metabolic activity of H6C7. *HC* was not cytotoxic to H6C7 even when the concentration of *HC* was as high as 250 µg/mL (*p* < 0.05). We conclude that *HC* effectively inhibits the growth of the four isolates without causing any harm to healthy cells. Figure 4C illustrates that *HC* concentrations at 125 µg/mL and below did not affect the cellular metabolic activity of H6C7. *HC* was not cytotoxic to H6C7 even when the concentration of *HC* was as high as 250 µg/mL. Therefore, we conclude that *HC* effectively inhibits the growth of the four isolates without causing any harm to healthy cells.

## Discussion

In recent years, the misuse of antimicrobial drugs has led to an increasing rate of clinical isolation of drug-resistant *Staphylococcus aureus* [26]. Methicillin-resistant *Staphylococcus aureus* (MRSA) can develop resistance to most antimicrobial drugs [27]. It is also well known that *Pseudomonas aeruginosa* is highly susceptible to drug resistance. And its resistance mechanisms are very complex. Clinically isolated carbapenem-resistant *Pseudomonas aeruginosa* (CRPA) generally develop concurrent resistance to antibiotics such as fluoroquinolones, aminoglycosides, and β-lactams [28]. Multi-drug-resistant *Pseudomonas aeruginosa* (MDRPA) can be resistant to multiple antibiotics [29]. Clinical patients are difficult to treat once infected with these bacteria. The development of new antibiotics is lengthy, expensive, and does not keep pace with bacterial resistance. The search for drugs that can replace antibiotics against bacteria has become a hot topic of study in recent years. In the present study, Compared with Met and Mer, HC treatment can significantly improve the infection of patients with DFU, and it could significantly reduce the expression levels of inflammatory factors (TNF-α and IL-6) in the serum of patients, and reduce the inflammatory response of patients. we investigated the inhibitory effect of *HC* on four isolates S08 (MSSA), R11 (MRSA), P10 (CRPA), and D22 (MDRPA) obtained from tissue and pus specimens isolated from patients with diabetic foot ulcers. The results of inhibition experiments showed that S08 (MSSA) was highly sensitive to *HC*, while R11 (MRSA), P10 (CRPA), and D22 (MDRPA) were moderately sensitive to *HC*. It is generally accepted that a drug can be recognized as a biocide if its MBC does not exceed four times its MIC. Our experimental results show that *HC* can inhibit the growth of bacteria in a certain concentration range, but it does not have a bactericidal effect. This suggests that we can use *HC* in combination with other drugs with bactericidal properties in the clinical treatment of patients with diabetic foot ulcers.

It is well known that methicillin and meropenem are two antibiotics commonly used in clinical practice to treat bacterial infections [30, 31]. Therefore, we investigated the effect of methicillin on the growth curves of S08 (MSSA) and R11 (MRSA). The results showed that methicillin could inhibit the growth of S08 (MSSA) to some extent, but not R11 (MRSA). Similarly, we investigated the effect of meropenem on the growth curves of P10 (CRPA) and D22 (MDRPA). The results showed that meropenem had no inhibitory effect on both strains of *Pseudomonas aeruginosa.* The above experimental results are consistent with those identified by Bruker mass spectrometer analysis. Interestingly, by analyzing the growth and time-kill curves we found that *HC* effectively inhibited the growth of the four isolates and even completely inhibited growth at their respective MIC concentrations. Therefore, we conclude that *HC* can effectively inhibit the growth of *Staphylococcus aureus* and *Pseudomonas aeruginosa*. This suggests to us that *HC* holds promise as a clinical treatment for diabetic foot ulcers. It is well known that, epithelial cells are the first line of defense of the body’s immune system against bacteria and bacterial products [32]. Therefore, we selected human normal pancreatic ductal epithelial cells (H6C7) to investigate whether *HC* would be cytotoxic to healthy eukaryotic cells. The results showed that *HC* effectively inhibited the growth of both bacteria without producing any toxic side effects on H6C7 cells. This demonstrates once again that *HC* may be a highly valuable new drug for the treatment of diabetic foot ulcers.

## Conclusion

In conclusion, *Houttuynia cordata* effectively inhibits the growth of *Staphylococcus aureus* and *Pseudomonas aeruginosa*. Moreover, cellular experiments demonstrated that *HC* is not cytotoxic to healthy eukaryotic cells. *HC* is highly likely to become a new clinical alternative to antibiotics for the treatment of diabetic foot ulcers. However, the mechanism of action of *HC* in inhibiting bacterial growth remains to be further elucidated. This study shows that HC treatment improves diabetic foot ulcer infections more effectively than methicillin and meropenem. It also significantly reduces serum inflammatory markers TNF-α and IL-6.

## Data Availability

No datasets were generated or analysed during the current study.

## Abbreviations

DM: Diabetes Mellitus
DFU: Diabetic foot ulcer
HC: Houttuynia cordata
MSSA: Methicillin-sensitive Staphylococcus aureus
MRSA: Methicillin-resistant Staphylococcus aureus
CRPA: Carbapenem-resistant Pseudomonas aeruginosa
MDRPA: Multi-drugresistant Pseudomonas aeruginosa
MIC: Minimum Inhibitory Concentration
MBC: Minimum Bactericidal Concentration
